# Supplementary material for: Driving under viral impairment: Linking acute SARS-CoV-2 infections to elevated car crash risks
Source: PLOS Glob Public Health. 2025 Apr 8;5(4):e0004420. doi: 10.1371/journal.pgph.0004420 (PMC11978055; doi:10.1371/journal.pgph.0004420)

18 Aug 2024

Dear Dr. Zimmerman,

Thank you for the opportunity to revise and resubmit my manuscript titled "Driving under viral impairment: Linking acute SARS-CoV-2 infections to elevated car crash risks" (PGPH-D-24-01012). I appreciate the thoughtful feedback provided by the reviewer and the editorial team. I have carefully considered all comments and have made substantial revisions to improve the manuscript. Below, I provide a point-by-point response to the reviewer's comments and detail the changes made to the manuscript.

Reviewer Comments:

1. **Thorough Literature Review:**
   - **Reviewer Comment:** The authors should structure a more thorough literature review, extending the existing insights to reflect the extensive research performed since the COVID-19 spread. The focus should be more on the traffic impacts and the origins of traffic crashes, highlighting that the increase in crashes is a combination of behavioral changes and primarily the reduction in traffic volumes. Relevant studies include:
     - Adanu, E. K., Brown, D., Jones, S., & Parrish, A. (2021). How did the COVID-19 pandemic affect road crashes and crash outcomes in Alabama?. Accident Analysis & Prevention, 163, 106428.
     - Sekadakis, M., Katrakazas, C., Michelaraki, E., Kehagia, F., & Yannis, G. (2021). Analysis of the impact of COVID-19 on collisions, fatalities, and injuries using time series forecasting: The case of Greece. Accident Analysis & Prevention, 162, 106391.
   - **Response:** I agree with the reviewer that a more comprehensive literature review was needed to place our study within the broader context of existing research on COVID-19's impact on traffic crashes. I have significantly expanded the literature review, incorporating the suggested studies and others that provide a more nuanced understanding of the behavioral changes and reductions in traffic volumes that contributed to crash outcomes during the pandemic. This revised section now better highlights the innovation of our study in linking acute SARS-CoV-2 infections to elevated car crash risks.

2. **Methodological Details:**
   - **Reviewer Comment:** The methodology should be extended to include the databases used, methods employed, and descriptive statistics of the datasets. Additionally, the authors should substantiate why Poisson regression was chosen.
   - **Response:** I have revised the methodology section to include detailed descriptions of the datasets used, data collection and the descriptive statistics of the variables considered. I have also provided a clear justification for the use of Poisson regression, noting its appropriateness for modeling count data, such as

the number of car crashes, and its ability to account for overdispersion. I have included supplementary files with the raw data.

3. **Discussion and Conclusions:**
   - **Reviewer Comment:** The discussion and conclusions should be strengthened in logical flow and discussed more thoroughly.
   - **Response:** I have restructured and expanded the discussion and conclusion sections to improve the logical flow and depth of analysis in tandem with introduction/background. I have also clarified the study's limitations and the need for further research, particularly in exploring the long-term effects of COVID-19 on cognitive function and driving safety.

Editor Comments:

1. **Raw Wastewater Data Collection and Analysis:**
   - **Editor Comment:** Please also provide details on your raw wastewater data collection and analysis, including sources of samples, lab procedures, and analytic techniques.
   - **Response:** I have not collected raw wastewater but have used CDC/HHS provided data as well as made this available in supplementary files as some of the older data may no longer be easily accessible online.

2. **Data Availability:**
   - **Editor Comment:** Please provide whatever data you can as supplementary files.
   - **Response:** I have ensured that all data underlying our findings are fully available as supplementary files.

I believe that these revisions address the concerns raised by the reviewer and the editorial team and substantially strengthen the manuscript. I appreciate the constructive feedback and hope that the revised version meets the standards for publication in PLOS Global Public Health. Please do not hesitate to contact me if further clarification or additional revisions are required.
Thank you for your consideration.

Sincerely,
Baran Erdik, MD, MHPA

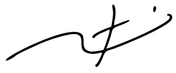

Supplement: Attachment — Submitted filename: response to reviewers.pdf [file pgph.0004420.s003.pdf]
