# Supplementary material for: Driving under viral impairment: Linking acute SARS-CoV-2 infections to elevated car crash risks
Source: PLOS Glob Public Health. 2025 Apr 8;5(4):e0004420. doi: 10.1371/journal.pgph.0004420 (PMC11978055; doi:10.1371/journal.pgph.0004420)
Supplement: Attachment — Submitted filename: response to reviewers_r2.pdf [file pgph.0004420.s004.pdf]

26 Jan 2025

Dear Dr. Nazif-Munoz,

Thank you for the opportunity to revise and resubmit my manuscript titled "Driving under viral impairment: Linking acute SARS-CoV-2 infections to elevated car crash risks" (PGPH-D-24-01012). I appreciate the reviewers' and the editors' thorough and constructive feedback on the manuscript. I have carefully considered all comments and have made substantial revisions to improve the manuscript. Below, I provide a point-by-point response to the reviewer's comments and detail the changes made to the manuscript.

Abstract and Methods Consistency

Reviewer Comment: In the abstract, the analysis is described as covering the period from 2020 to 2023 (lines 19–20, page 2). However, in the Methods section (page 8, line 143), data collection is stated to have occurred between March and May 2024. Please clarify the exact data period used in the study.
Response: I have clarified that the data underlying the statistical analysis covered the period from 2020 to 2023. Data collection was performed in 2024 to allow for finalization and completeness. This distinction is now explicitly stated in both the abstract and the Methods section for consistency.

Materials and Methods

Reviewer Comment: Specify the exact number of states included in the analysis instead of referring to "several" states.
Response: The manuscript now specifies that the analysis included seven states (Connecticut, Florida, Georgia, Iowa, Massachusetts, Texas, and Washington).

Reviewer Comment: Elaborate on the extent to which crude and refined wastewater metrics were utilized.
Response: Commentary has been added in the Methods section, elaborating on how crude and refined wastewater metrics were utilized and validated, supported by references to previous literature.

Reviewer Comment: Include explicit references for the sources of information described in the Methods section to ensure transparency.
Response: References have been added for all data sources, including state Department of Transportation repositories and CDC metrics.

Results Section

Reviewer Comment: Ensure there is no repetition in the text. For example, the wording in lines 181–185 (page 10) is repeated.
Response: Repetition in the specified section has been removed to enhance clarity and conciseness.

Reviewer Comment: Correct the duplication of Table 2 to avoid confusion.
Response: The duplication of Table 2 has been resolved, and the results section now refers to the correct and unique tables.

Terminology

Reviewer Comment: Avoid using the term "accidents" and consistently use "crashes" throughout the manuscript.
Response: The term "accidents" has been replaced with "crashes" consistently throughout the manuscript to align with accepted terminology.

Methods Section

Reviewer Comment: Provide a detailed description of the Poisson model used, including whether fixed or random effects were applied, and justify the choice in light of state-level analysis.
Response: The Methods section now includes a detailed explanation of the Poisson regression model, including the application of fixed effects to account for state-specific variability and a justification for this choice.

Reviewer Comment: Describe how overdispersion was assessed and explain why a Negative Binomial model was not used.
Response: Overdispersion was assessed using the Pearson dispersion statistic. As the overdispersion was minimal (dispersion statistic close to 1), the Poisson model was deemed appropriate. This justification has been added to the Methods section.

Reviewer Comment: Move the description of the heat map from the Results section to the Methods section and clarify how it was constructed and used to identify variables for the models.
Response: The heat map description has been moved to the Methods section and expanded to clarify its role in identifying and excluding highly correlated variables.

Reviewer Comment: Revise the equation to properly include the log of the population as an offset, and ensure the outcome is clearly described as a log-transformed variable.
Response: The equation has been revised to explicitly include the log of the population as an offset, and the outcome is described as a log-transformed variable.

Reviewer Comment: State the software used to perform the analyses.
Response: The Methods section now specifies that all analyses were performed using SAS version 9.4 (SAS Institute Inc., Cary, NC).

Discussion Section

Reviewer Comment: Revise the statement in line 269 (page 15) to avoid conflating fatalities and crashes, as these are distinct outcomes. Ensure the discussion aligns with the specific outcome being analyzed.
Response: The statement has been revised to distinguish between fatalities and crashes clearly. The discussion has been aligned to focus on the study's specific outcome, namely, the association between acute COVID-19 infections and car crashes.

References

Reviewer Comment: Include and properly cite the following reference, which was mentioned in your discussion:
R.R. Knipling, "Crash Heterogeneity: Implications for Naturalistic Driving Studies and for Understanding Crash Risks," Transp. Res. Rec., 2663 (2017), pp. 117-125, 10.3141/2663-15.
Response: This reference has been added to the discussion and included in the reference list.

I believe that these revisions address the concerns raised by the reviewer and the editorial team and substantially strengthen the manuscript. I appreciate the constructive feedback and hope that the revised version meets the standards for publication in PLOS Global Public Health. Please do not hesitate to contact me if further clarification or additional revisions are required.
Thank you for your consideration.

Sincerely,
Baran Erdik, MD, MHPA
